# A Global Voting Advice Application for the US Election Aimed at Foreign Audiences

**DOI:** 10.1038/s41597-025-06333-6

**Published:** 2025-11-28

**Authors:** Tom W. Etienne, Neil V. Bennett, Lily Markis-McLean, Richard. A. Furstein, Yordan Kutiyski, Jeroen van Lindert, Andrew C. Pasquier, Oscar Moreda Laguna, Jan Philipp Thomeczek, Andre P. M. Krouwel

**Affiliations:** 1 University of Pennsylvania, Philadelphia, PA USA; 2 Kieskompas – Election Compass, Amsterdam, Netherlands; 3 University of Potsdam, Potsdam, Germany; 4 VU University Amsterdam, Amsterdam, Netherlands

**Keywords:** Politics, Communication

## Abstract

Billions of people outside of the US hold a vested interest in the outcome of US presidential elections, despite not being allowed to vote. We developed 9 Voting Advice Applications (VAAs) for the 2020 elections and 5 VAAs for the 2024 elections in partnership with leading newspapers across Europe, Asia, and Oceania. Across these two elections, the VAA tools offered 724,294 users from 187 countries an issue-based comparison with Biden and Trump in 2020, and with Trump and Harris in 2024. Users answered demographic questions, the issue statements, and additional attitudinal items. These issue statements covered both domestic topics and topics of the US’ foreign policy. The candidates’ positions were coded using robust primary and secondary sources on 29 (2020) and 62 (2024) issue statements. In this paper, we describe the candidates’ placement data as well as the user data generated by these VAAs, offering an unprecedented opportunity to analyze global perceptions of US politics and the election’s far-reaching influence on international relations.

## Background & Summary

The US presidential election is arguably the most salient foreign election worldwide. Beyond the US population, billions of people across the globe are impacted by the outcome of who wins the White House. The policies of the US president influence global security, trade, climate policy, and international cooperation, making the election’s implications far-reaching. Despite its importance, foreign perspectives on the US election remain understudied, even as scholars have come to grapple with the relevance of such perceptions in understanding the stability of US hegemony and the diffusion of ideology across borders. Collecting data on foreign opinions regarding the US election allows us to better understand how international audiences perceive American democracy, its candidates, and their policy positions.

To capture these perspectives, we developed Voting Advice Applications (VAAs) for the 2020 and 2024 US elections tailored to an international audience. These tools are available at usa2020.electioncompass.org and usa2024.electioncompass.org, respectively. VAAs are interactive tools that help users identify which candidate aligns most closely with their policy preferences. Traditionally used for national elections, VAAs also serve as valuable research instruments for gauging public opinion. Our VAAs present users with a series of Likert-scale issue statements reflecting key campaign issues, often with a particular international angle. The tool then compares their responses to the positions of Biden and Trump, and Trump and Harris respectively, calculating the proximity between the candidates and the user. The tools were developed by researchers at the Dutch Kieskompas – Election Compass research institute in collaboration with scholars at the University of Pennsylvania and the VU University Amsterdam. They were funded and disseminated by major news outlets across a range of countries as well as by the University of Amsterdam.

Our contribution is comprised of four main datasets. For both the 2020 and 2024 elections, we coded the respective candidates’ positions on 29 and 62 statements, respectively. Each candidate position includes a 5-point Strongly Agree to Strongly Disagree scale point, which is supported by up to three sources. In addition, again for both elections, we present the responses of 571.624 and 152.670 users, respectively. The 2020 tools were disseminated in Belgium, Germany, Denmark, France, the Netherlands, Romania, Bulgaria, and the US. While our ‘base’ VAA was tailored to an American audience, each disseminated version was customized with questions specifically relevant to the international audience it served. The 2024 tools were disseminated in Belgium, India, and by an Asia-wide newspaper, in addition to the tool tailored to the US. In total, the tools were accessed by users in 187 countries. The respective media partners and user totals are indicated in Table 1.

**Table 1:** Overview of countries and regions where the VAAs were disseminated, by which media outlet (if any), and total number of users.

Country/region	Media partner	2020 users	2024 users
Asia-wide	The Diplomat	—	1,449
Belgium (Flanders)	Het Laatste Nieuws (HLN)	227,464	126,626
Belgium (Wallonia)	7SUR7	31,584	20,474
Bulgaria	Blitz.bg	3,390	—
Denmark	B.T. Nyheder	90,233	—
France	20 Minutes	27,216	—
Germany	Jetzt.de	171,046	—
India	The Hindu	—	1,848
The Netherlands	Algemeen Dagblad	13,438	—
Romania	No media partner	6,797	—
United States	No media partner	456	2,273
Total		571,624	152,670

This dataset has the potential to inform policy debates and enhance academic understanding of international electoral perceptions and their downstream consequences. Specifically, it allows researchers to explore how foreign publics evaluate US candidates and their policy stances, shedding light on perceptions of US leadership and broader patterns of ideological diffusion across different regions and political contexts. Ultimately, these insights can be leveraged by policymakers, media organizations, and academics to refine international engagement strategies, assess the global reception of US foreign policy, and deepen our understanding of the international ramifications of US electoral outcomes.

## Methods

Before detailing the data generation process, we briefly discuss the dual role of voting advice applications (VAAs) as both informational tools for users and instruments for collecting public opinion data. We then outline the development of the issue statements and the qualitative coding of the candidates’ positions. Finally, we describe the process by which the user data was generated through interaction with the VAAs.

Voting advice applications are online tools designed to provide voters with information regarding substantive overlap with parties or candidates running in a particular election. They emerged in the 1990s with the advent of the internet, and are now commonplace in many multiparty systems around the world^1^. In some countries, like the Netherlands, certain VAAs are consulted by up to half of the electorate^2^. However, these tools do not attract random subsets of the population, often leading to samples with an overrepresentation of young, highly educated males^3,4^ without an immigration background and a disproportionate level of political interest^5^. While such differential consultation may have attenuated over time^6^, it remains heavily conditioned by the level of internet penetration and usage in a country^7^. In addition, these tools are exposed to a large number of designer degrees of freedom. Both the wording of issue statements^8,9^ and the selection of included issues^10^ can affect the results that the tools provide. Subsequently, the particular algorithms that are used to match users with candidates based on their responses can introduce another layer of variability^11,12^. We should therefore understand the capacity of VAAs to provide voting advice in the context of their design decisions and the tools’ reach.

Aside from providing information to voters, these tools are unique data collection devices. The placements of the parties or candidates have been used in electoral analysis^13^, presenting insights into the estimated policy positions of these entities relative to each other^14^. The combination of public opinion data and party placements has been used in the study of electoral congruence^15^. Perhaps the largest advantage of VAAs over alternative data collection methods for public opinion data is their large reach. Like other nonprobability samples, VAA data faces selection bias risks, which occurs when opt-in occurs at differential rates^16,17^. Nevertheless, both the data and the panels that result from these VAAs have frequently been used in academic publications^18–20^. Given the appropriate statistical methods, VAA data can reliably be used for estimating voter policy positions^21^, providing evidence for the validity of VAA data as instruments for public opinion research.

The tools that collected the user data as presented here are designed by the Amsterdam-based *Kieskompas - Election Compass* research institute, which develops the second-most visited VAA during Dutch elections, and which has developed VAAs in over 30 countries. Previous work has outlined the approach that the research institute takes with regards to the development of their VAAs^22,23^. The following two sections provide a detailed account of the development of the VAAs for the 2020 and 2024 US elections.

### Candidate positions

The first step in designing the candidate position data involves issue mapping and the drafting of issue statements. This process begins with the creation of a longlist of politically salient topics that are particularly relevant to foreign audiences. Coders then refine this list by merging overlapping issues, excluding those deemed non-salient, and ultimately producing a finalized shortlist of statements for inclusion in the VAA. For each selected issue, a corresponding statement is formulated. These statements are crafted to be as unambiguous as possible^9^, avoiding unnecessary quantification or qualification^24^, and are designed to capture ideological positions rather than specific policy proposals^25^. At this stage, each statement is also categorized as reflecting a culturally liberal, culturally conservative, economically left, or economically right position, aiming for a roughly equal number of each pole per dimension^8^. This careful formulation process ensures that the issue statements are both accessible to users and analytically useful for subsequent coding and analysis, producing 29 statements in 2020 and 62 statements in 2024.

Table 2 lists all statements used in the 2020 and 2024 VAAs across all newspapers. Table 3 lists all statements that the candidates were positioned on, but did not feature in all newspapers in their respective cycles. In the few cases where a statement appeared in only one publication, the name of that publication is indicated. Many statements were reused between 2020 and 2024, although at times with slight modifications as indicated. In 2020, 29 statements were coded in total, 26 of which were ultimately included in the VAA. The 2024 iteration drew from a larger set of coded statements (62). The VAAs distributed in Belgium and the United States included 25 statements, while the versions published in *The Diplomat* and *The Hindu* each had 30 statements. These additional statements included some of those developed during the initial issue formulation and placement coding process, and some developed specifically to address issues relevant to the audiences of these publications.

**Table 2:** Overview of the statements that are included in the disseminated VAAs.

Statement text	Code 2020	Code 2024
Access to abortions should be restricted	021	
Climate change is an existential threat		003
Combatting climate change requires international cooperation		002
Discrimination against transgender people should be made illegal	010	
Federal troops should be sent into cities to restore law and order during protests	027	056
Higher education needs to be made less expensive	011	
Immigrants should be deported if they commit misdemeanor offences	025	
Israel and Palestine should negotiate a ceasefire agreement		025
Israel has a right to annex territory in the West Bank	014	023*
It is important to transition to clean energy as quickly as possible		005
Labor unions hurt the economy	007	016
Mail-in ballots will lead to election fraud	017	
Men and women have different roles in society		008
NATO is obsolete	012	
Prisons should be operated by the government, not by private corporations	029	
Private money should be eliminated from elections	015	
Regulation of big tech harms economic competition		012
Russia is interfering with the US election in favor of Trump (2020) Russia interfered in the 2020 US election in favor of Trump (2024)	013	046*
Russia is a threat to world peace		031
Schools should be able to ban books on certain topics including race, gender, and sexuality		017
Social media platforms should ban right-wing extremist posts, even if it limits freedom of speech	002	006
Stricter gun laws will make the US safer		053
Taxes on the wealthy should be increased (2020) Taxes on the wealthy should be increased in the US (2024)	006	015
The Black Lives Matter movement is a justified way to combat racism	003	
The candidates reject the election result when they believe that election fraud took place, even when those claims are not proven		051
The death penalty should be abolished	028	043
The government has a responsibility to ensure all Americans have health insurance	022	
The mainstream media is misrepresenting facts to indoctrinate the public with liberal ideas	004	
The right to abortion should be protected across the entire US		034
The US federal government has handled the COVID-19 pandemic well	020	
The US government has a responsibility to ensure all Americans have health insurance		036
The US has a duty to come to Taiwan’s aid if it is attacked by China		020
The US needs a president that gets things done, even if that means breaking the rules (2020) The US needs a president that gets things done rather than sticks by the rules (2024)	018	050
The US should come to the aid of NATO allies when they are attacked, even if they do not meet military spending targets		028
The US should complete the wall along the border with Mexico	024	040
The US should do more to help the Palestinian people		024
The US should focus on issues at home before it gets involved in other countries’ wars		033
The US should increase tariffs on Chinese imports across the board		013
The US should increase the deportation of illegal immigrants		038
The US should rejoin the Paris Climate Agreement	001	
The US should rejoin the World Health Organization (WHO)	023	
The US should seek diplomatic ties with North Korea, even if they continue their nuclear program for the time being		029
The US should send Ukraine military aid for as long as it needs to win the war against Russia		030
There exists systemic police violence against minorities in the US		055
There should be a ban on assault weapons	026	054
To protect its economy, the US should impose a universal tariff on imports		032
Treaties that promote free trade are good for the economy	008	062^†^
Wearing a mask should be mandatory in all public spaces across the country	019	

Statement codes require prefixes uspr20_ and uspr24_ respectively. Statements denoted with * feature only in the Diplomat in 2024, and with ^†^ only in The Hindu in 2024.

**Table 3:** Overview of the statements that are not included in all disseminated VAAs.

Statement text	Code 2020	Code 2024
American families should be guaranteed childcare		019
Babies born in a country should automatically be granted citizenship, regardless of their parents’ citizenship status.		042
Breaking up the Iran nuclear deal made the world a safer place		022*
Corporations should not fund election campaigns	016	
Discrimination against transgender people should be illegal		011
Imposing trade tariffs is a good way to protect the US economy	009	
In the US, the death penalty should be imposed for a wider range of crimes		044^†^
India should condemn the Russian invasion of Ukraine		060^†^
Marijuana should be legal across the entire country		052
Migrants who came to the US illegally as kids should be able to remain in the country		041
More countries, including India, should impose sanctions on Russia in response to its actions in the Russia-Ukraine war		061
Sanctions on Iran are more effective at containing the country than diplomatic pressure		021
Serious natural disasters are a sign of climate change		004
The 2020 US presidential election was stolen		045
The best way to solve the housing crisis is to incentivize private companies to build more housing, rather than through direct government intervention		014
The government should provide health insurance to those who cannot afford it		037
The government should recognize only two genders		010
The mainstream media is “fake news”	005	
The mainstream media is trying to indoctrinate the public with liberal ideas		007
The most effective way to address the opioid crisis is through strict law enforcement measures rather than expanding access to treatment and prevention programs		035
The powers of the US federal government are currently excessive and must be restrained		047
The United States should suspend military aid to Israel in response to its conduct in the war with Hamas in Gaza		058^†^
The US government is in need of an overhaul to rid it of rogue bureaucrats		048
The US government should forgive student loan debt		018
The US president should have immunity for any crimes committed during their term		049
The US should advocate for a two-state solution in the Israel-Palestine conflict		026*
The US should remain a member of the Paris Climate Agreement		001
To counter the rise of China, the ‘Quad’ partnership between the US, India, Japan, and Australia should evolve into a formal security alliance		059^†^
To ensure diverse student bodies, American universities should be allowed to take ethnicity into account when admitting students		009
Undocumented immigrants should be deported if they commit misdemeanor offences		039
US allies should pay more for a guarantee of US military support in a conflict		057*
Without drastic investments, the US military is falling behind those of other countries		027

Statement codes require prefixes uspr20_ and uspr24_ respectively. Statements denoted with * feature only in the Diplomat in 2024, ad with ^†^ only in The Hindu in 2024.

The two major-party candidates in both elections are positioned on all of the above statements. The placement coding process includes multiple steps to ensure that coders independently reach conclusions about how to position the candidates against each of the statements on the shortlist. In 2024, two coders independently completed the coding process, while a third coder resolved any discrepancies between the initial two coders. The intercoder reliability between the two initial coders was 0.78 (weighted Cohen’s kappa, ICC, and Krippendorf). In 2020, due to more limited funding, two coders evaluated only a subset of the statements. After reviewing the level of agreement and reliability within this subset, it was determined that a single coder could reliably code the remaining statements. During the placement coding process, coders find authoritative sources that clearly indicate the candidate’s position on a given issue. Sources are valued hierarchically, with official candidate platforms and candidate speeches and interviews preferred. We consider such sources to be primary sources. If these are not available for a given statement, coders then look for related positions or reliable media reports that either indicate the candidate’s position or allow us to infer their position with a high degree of confidence. Third, coders looked at candidate voting records. It should be noted that a significant proportion of Trump’s 2020 positions were sourced from Twitter (now X) given his heavy reliance on the platform while campaigning. All of these sources were weighted heavily based on their recency, with particular caution applied to evidence from previous administrations, which was considered less indicative of current positions. When sources provide conflicting information about a candidate’s position, coders evaluate them based primarily on recency and the hierarchy of sources. For each statement, we require a minimum of one source and a maximum of three to justify the coder’s positioning of the candidate. The statements feature five-point Likert scales and include a ‘No Opinion’ option. For a candidate to be coded as ‘Strongly Agree’ or ‘Strongly Disagree’ with a statement, we require evidence of unqualified support or opposition to the issue, respectively. Candidates can also be coded as ‘Agree’ or ‘Disagree’ in instances where they voice conditional agreement or disagreement. Candidates who offer highly nuanced positions are coded as ‘Neither Agree Nor Disagree,’ while candidates who have not voiced any position are coded as ‘No Opinion.’ Statements where either one of the candidates is coded as having ‘No Opinion’ will not be featured in the VAA, but we retain those statements in the candidate placement data we share.

**Fig. 1:**
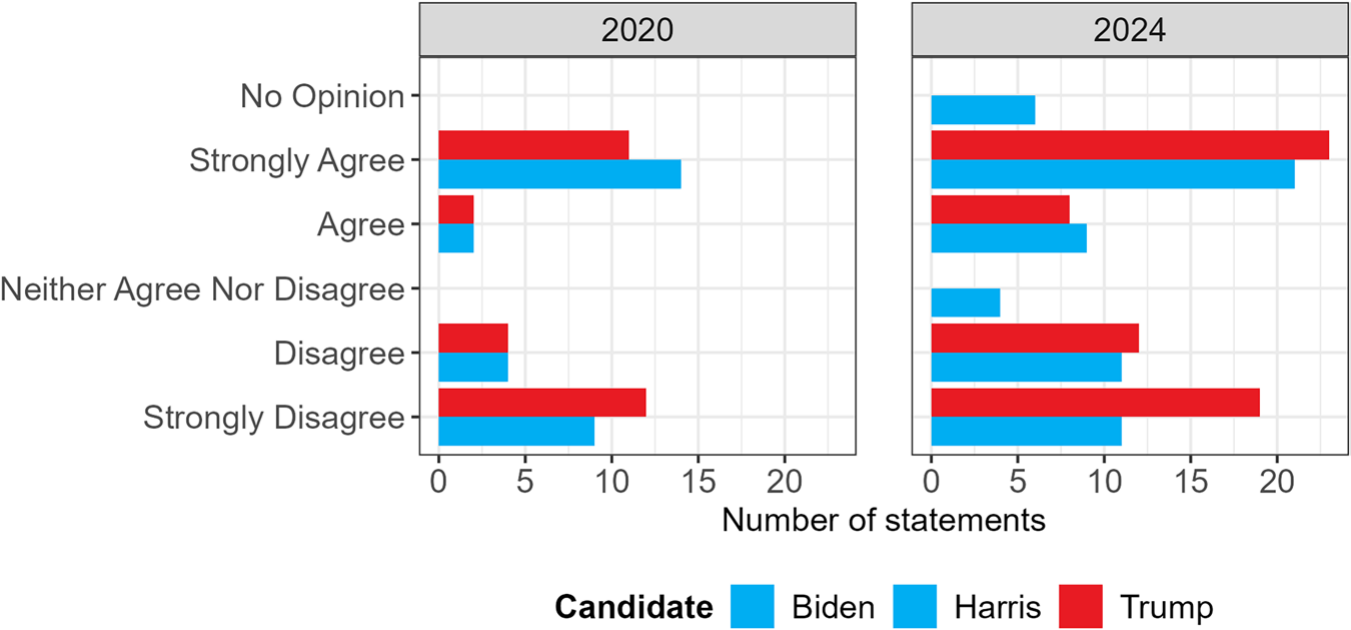
Histogram of each candidate’s positions on the Likert scale, by year.

**Fig. 2:**
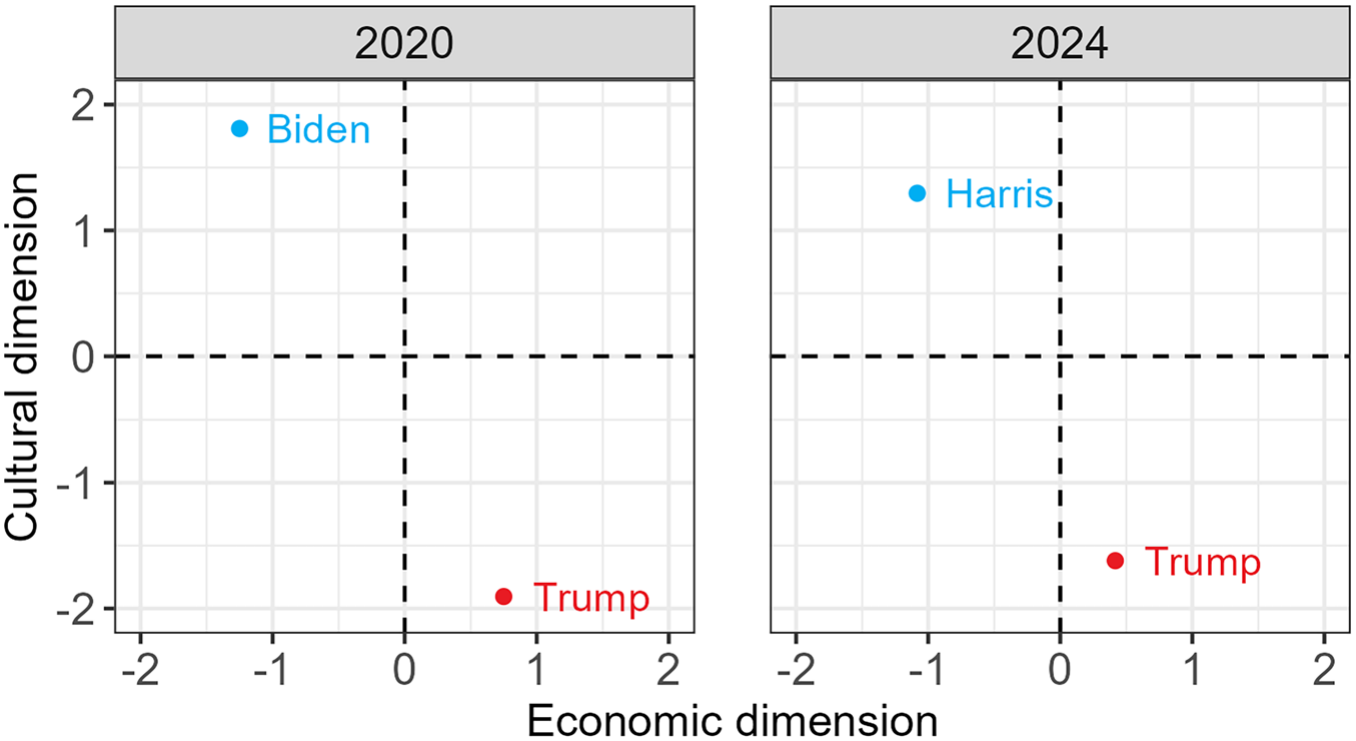
Each candidate’s position in the political landscape as resulting from placements on the statements.

To ensure clear differentiation between the two candidates in the VAA, issues were selected and worded to maximize the contrast in their coded positions. This resulted in a higher proportion of placements using the extreme ends of the Likert scale as shown in Fig. 1, and a position in the political landscape at the respective edges as shown in Fig. 2. Walgrave^10^ reminds us that the selection of statements in a VAA can greatly impact its results, cautioning against the interpretation of the candidates’ positions in the political landscape as an ideal point on an ideological dimension. We include these figures simply as a point of reference.

### User data

This section starts by outlining the VAAs’ dissemination strategy during the election campaigns. It then details the data-generating process, after which it discusses the reach and make-up of the data.

**Fig. 3:**
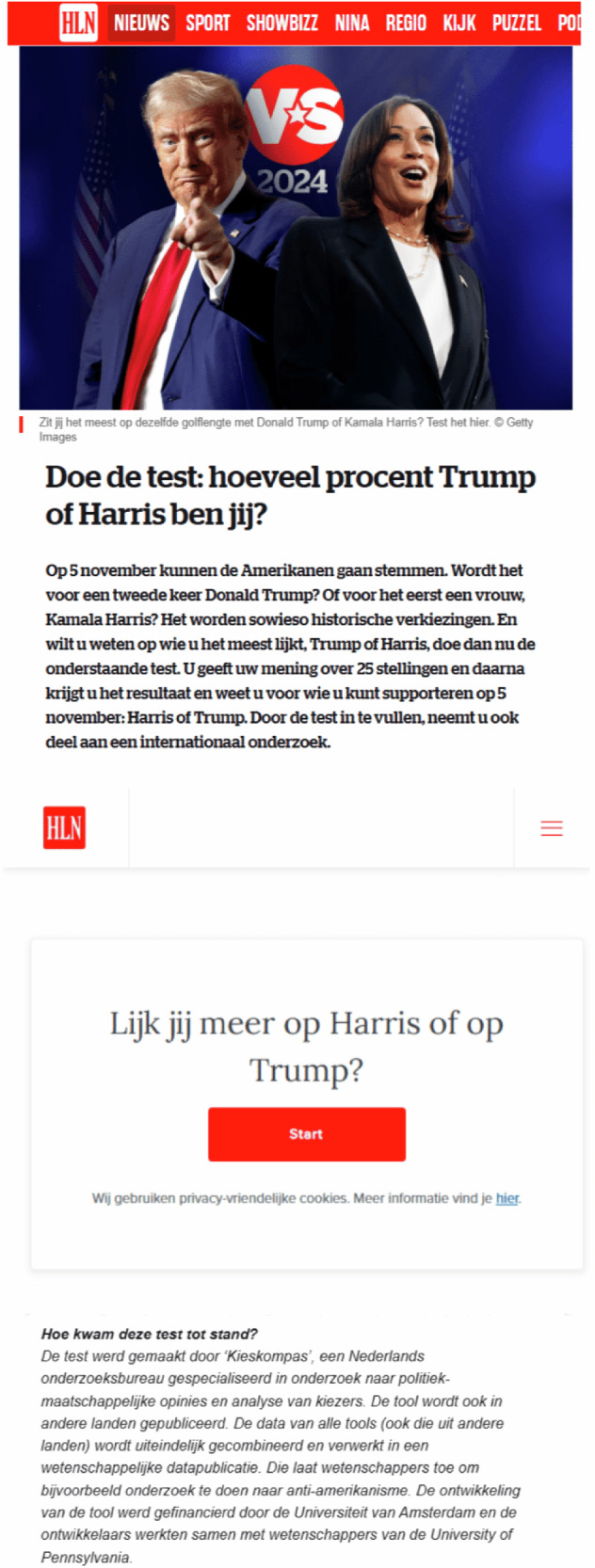
Example of integration of the VAA into a media outlet’s article. Shown here: HLN (2024).

The VAAs were primarily disseminated through major national media outlets in selected countries. Some of these outlets commissioned the tool directly from the developing research institute, while others translated the tool and received permission to host it on their platforms through an embedded iframe. We consider the outlets that incorporated the tool into their own platforms to be media partners, as detailed in Table 1. Figure 3 illustrates how the tool was embedded via an iframe within an article by *HLN*^26^. All participating media partners integrated the tool in a similar manner, except *The Hindu* who featured the tool as a standalone popup article on its homepage in 2024. Several partners also published follow-up articles analyzing aggregated user responses, sometimes embedding the tool again within their coverage. In addition, there were media outlets that simply reported about the tool that was available in their country, without the inclusion of such an iframe. While the media partners tended to be the largest source of traffic, the German tool in 2020 for instance attracted substantial traffic from outlets like *T-Online* who simply reported about the VAA. In 2024, the project was awarded the University of Amsterdam’s Alumni Impact Prize, which provided additional funding to support dissemination beyond European media. The tools for the 2020 and 2024 elections remain accessible via usa2020.electioncompass.org and usa2024.electioncompass.org, respectively.

**Fig. 4:**
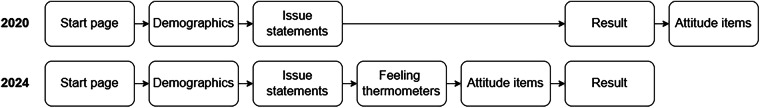
Flowchart of VAA content.

Users go through a series of pages to arrive at the result page of the tool, as indicated in Fig. 4. After clicking ‘Start’ on the start page, users move through a series of demographic questions. These include age, gender, education, geographic location, and, in some 2024 cases, which party the user voted for in their last national election. Users proceed to the issue statements, which are shown one-by-one with the Likert scale below them. Users can return to update their answers, indicate that they have no opinion on the issue at hand, or skip the issue altogether. At this point, the 2020 and 2024 tools diverge slightly. The 2024 tools display a series of feeling thermometer items on 11-point scales, asking how favorably users feel about the presidential candidates, the two political parties, the American people, and the governments of the United States, China, and Russia. These questions are also optional, and users are able to select either ‘No opinion’ or ‘Prefer not to say’ for each of them. The software did not allow randomizing the order of these items. The 2024 tools ask another set of attitudinal questions before displaying the results of the tool. The attitude items ask the user about their economic and cultural attitudes, presidential candidate voting preference, and attitude toward the United States. These items are optional in both VAAs. Similar items were asked in 2020 after displaying the results, leading to lower item response rates. The 2020 tools move straight into the result pages after users answered the 26 issue statements. The 2020 tool shows three result pages: a ranking of both candidates, indicating a percentage agreement score; a two-dimensional landscape, akin to the ones shown in Fig. 2, which now includes the position of the user; and a page which details both candidates’ positions as well as the sources that underpin these positions. The 2024 tool shows only the first and last of these result pages, leaving out the political landscape due to the limited number of included items pertaining to the economic dimension. Users have the option to filter their results by the broad issue statement themes. All of these different pages in the tools collect users’ responses, producing a rich public opinion dataset.

Note that the 2020 French tool, disseminated by 20 Minutes, linked immediately to the issue statement page so that a large number of users did not see the demographic background items. To account for this, the same background items were added in the attitude items shown at the end of the tool, and subsequently entered into the demographics data.

In addition to the data entered by users, or calculated on their responses, the tools collect certain fields automatically.

**Fig. 5:**
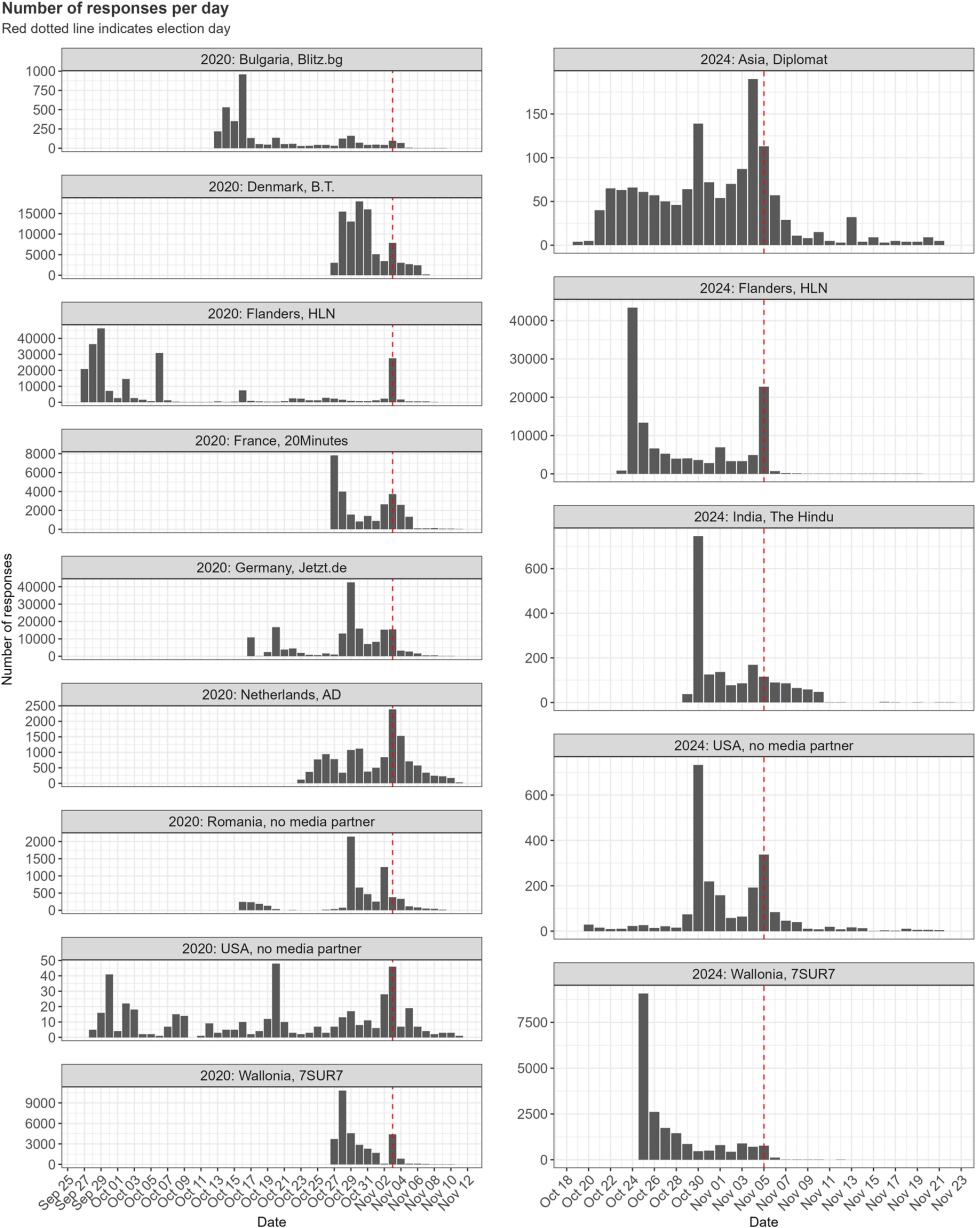
Histograms of user numbers, by tool and year.

We discuss some descriptives of interest below to provide a sense of the data. First, in Fig. 5, we show user numbers by day for all of the tools. It is clear that tools attract most users on and immediately after the day that their respective media partners launched the tool. Occasional peaks in the run-up to the election tend to be attributable to additional media attention, in nearly all instances by the disseminating outlet itself. In the days immediately before and on election day itself, numbers also increase relative to previous days. We include data for a number of days after the election to allow for analysis of changing patterns.

**Fig. 6:**
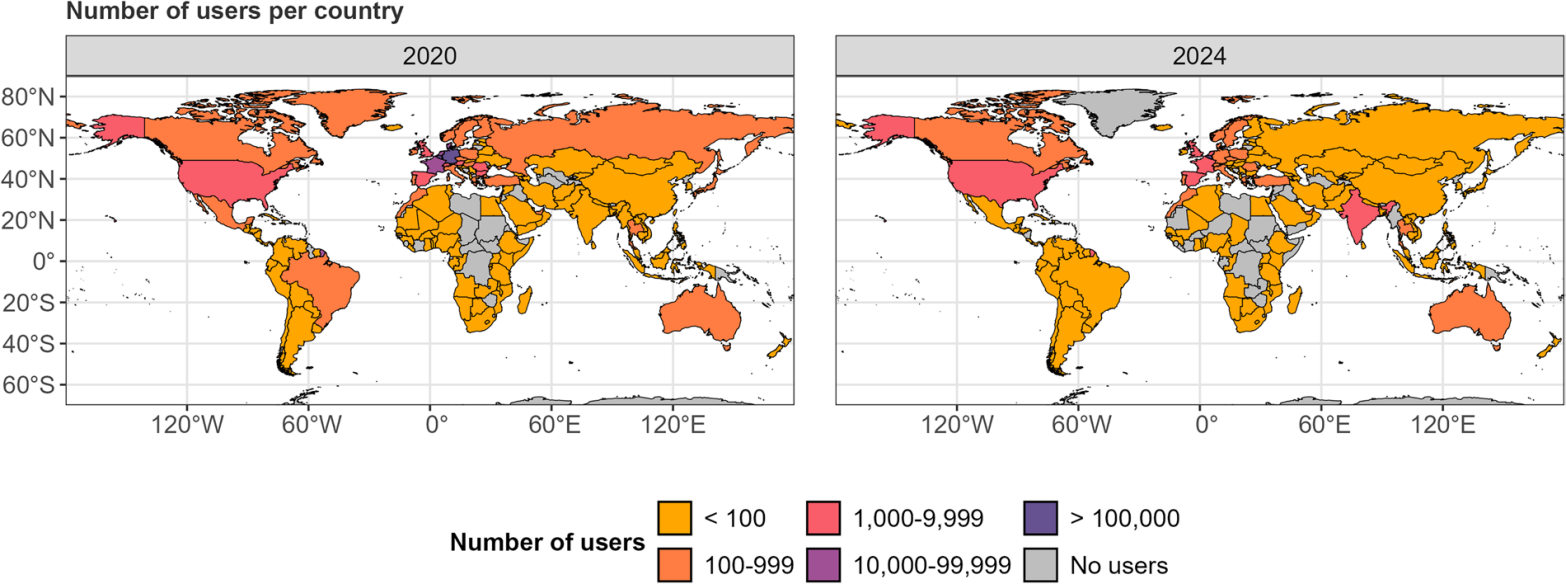
Map of users’ origin.

Second, while most responses originated from the countries in which the VAAs were disseminated, responses came in from all around the world. Figure 6 displays which countries’ responses were registered from (i.e., not where users indicated to live).

**Fig. 7:**
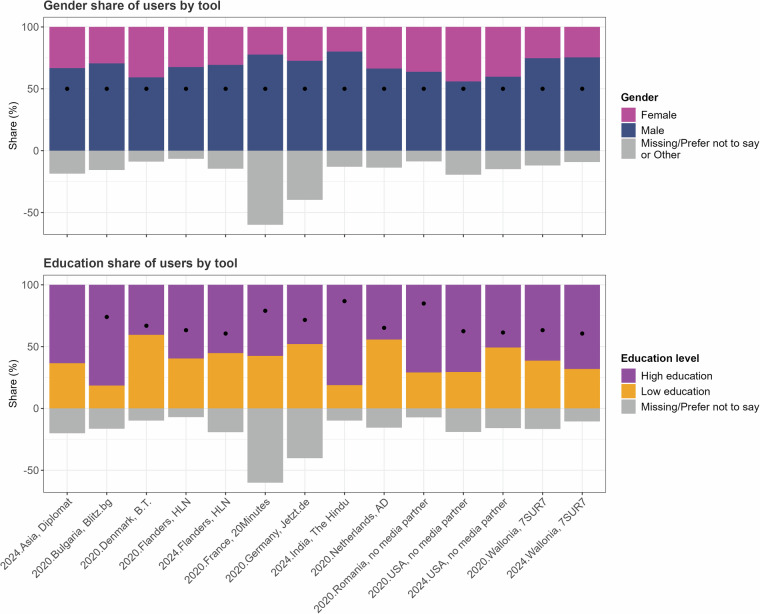
Demographic distribution by tool and year, for gender and education.

Third, we provide some indication of the representativeness of the samples. Since users were not invited to participate, but rather self-selected into participation with no intervention on the part of the authors aside from the collaboration with the media partners, the samples display biases commonly seen in nonprobability samples, particularly those stemming from VAAs^3,4,6^. As Fig. 7 shows, all tools know a gender bias with men being overrepresented (the black dot indicates the 50% proportion). The Male and Female bars are rescaled to 100% for straightforward interpretation, whereas the negative grey bars indicate the total proportion of respondents answering “Other,” “Prefer not to say,” or those with missing gender data. In addition, the tools were overwhelmingly consulted by users who enjoyed higher education. The dots on the bottom facet indicate the education levels in the population of the respective countries, as drawn from UNESCO data. There is thus an overrepresentation of higher-educated users and male users.

All data were collected in accordance with the European General Data Protection Regulation (GDPR) as certified by *Privacy Certified*. No personally identifiable data was collected. Data collection was approved by the VU University Amsterdam (2024-10-15-168). By commencing the use of the tools, all respondents consented to participation and agreed to the cookie and privacy policy (to be found at kieskompas.nl/nl/policies), which includes consent for anonymous data sharing.

## Data Records

We describe 4 datasets and 3 other files that are made available on the Inter-university Consortium for Political and Social Research (ICPSR) database [238229]^27^. The first two datasets comprise the candidates’ placements on 29 statements in 2020 and 62 statements in 2024. The second two comprise the user data, reflecting 571,624 users in 2020 and 152,670 users in 2024. We also include a codebook for these four datasets. In addition, we include an overview of all statements between both election cycles, and which VAAs these statements were included in, as well as a file with code to replicate the tables, figures, and numbers in this manuscript, including code that follows our recommended usage notes.

The placement data includes two files, named ‘candidate_placements_2020.csv’ and ‘candidate_placements_2024.csv’. Each file is structured identically. The first column ‘statement_code’ has a unique identifier for the issue statement, followed by the second column ‘statement_text’. Note that these do not uniquely identify the row, which is uniquely identified in combination with the third column ‘candidate’, indicating the candidate being placed against a given statement. In 2020, these are Biden and Trump; in 2024, these are Trump and Harris. The following column ‘candidate_position’ indicates their assigned position. Subsequent columns contain the sources used to underpin the candidates’ position. Each position has up to three sources, each consisting of three columns: ‘source_1_origin’, ‘source_1_url’, and ‘source_1_excerpt’. Each row furthermore specifies whether we consider a statement to address a cultural or an economic issue, and which direction the statement is scaled under ‘statement_scaling’. Statement scaling takes values L for economic left, R for economic right, P for culturally liberal (progressive), and C for culturally conservative. Lastly, ‘in_vaa’ indicates whether a statement was included in the VAA, and, if so, in which one. Table 4 lists these variables.

**Table 4:** Codebook for the candidate placement datasets.

Variable name	Description	Values
statement_code	Unique identifier for each issue statement (not unique by itself).	
statement_text	The full wording of the issue statement.	
candidate	The candidate being placed	2020: Biden, Trump; 2024: Harris, Trump.
candidate_position	The assigned position of the candidate on the issue statement.	Strongly Disagree, Disagree, Neither Agree Nor Disagree, Agree, Strongly Agree, No Opinion
source_1_origin	Origin/source of the first justification for the candidate’s position.	
source_1_url	URL of the first source.	
source_1_excerpt	Quoted excerpt from the first source.	
source_2_origin	Origin/source of the second justification. (Optional)	
source_2_url	URL of the second source. (Optional)	
source_2_excerpt	Quoted excerpt from the second source. (Optional)	
source_3_origin	Origin/source of the third justification. (Optional)	
source_3_url	URL of the third source. (Optional)	
source_3_excerpt	Quoted excerpt from the third source. (Optional)	
statement_scaling	Indicates the dimension and direction in which the statement is scaled.	L, R, C, P
in_vaa	Indicates whether the statement was included in the VAA (and if so, in which version).	none, all, diplomat, india

In addition, we share two user data files, corresponding to users’ responses from the 2020 and 2024 surveys. These files are named ‘user_data_2020.csv’ and ‘user_data_2024.csv’ respectively. The files’ first column ‘tool’ identifies the tool source. The second column ‘token’ identifies the user with a unique alphanumerical combination. The column ‘language’ indicates the language in which the tool was completed, although users could also rely on applications like Google Translate to translate the content. Such translations would not be reflected in this column. The next two columns ‘date’ and ‘session_start’ indicate the day and the precise time that a user visited the tool. The ‘before_after_election’ converts the start time to whether the tool was filled out before or after the first election results came out. The next few variables pertain to how long users spent answering the tool in seconds, and how many seconds of those were spent on the issue statements. The data includes columns on what percentage of the statements were answered, and how many statements were answered in the same way, as well as what proportion of answered statements this entails. The next two variables indicate what device type was used to access the tool, and whether this was a touch device or not. The ‘continent’, ‘country’, and ‘subdivision’ variable contain automatically collected geographic data. As detailed under Usage Notes, we then include a number of variables that the user may find useful in filtering records. The following columns provide the entered demographic information by the respondent and are denoted with the prefix “bgq.” The next set of variables pertain to the user’s answers to the issue statements. These columns are named after the placement data’s ‘statement_code’ values. In 2020, the result variables follow. There are three columns with the prefix “pct”, which, based on the respondent’s answers to the issue statement questions, provide their percentage alignment with Joe Biden and Donald Trump, and then their highest agreement candidate. In addition, the 2020 data contains the user’s resulting ‘position_x’ and ‘position_y’. The 2024 data, before the percentage agreement result variables, includes the 11 feeling thermometer variables, denoted with the prefix “fts”. After that, eight columns describe the respondent’s attitudes toward a range of variables and are denoted with the prefix “att.” The 2020 data includes these additional attitudinal items after the result variables, to remain true to the ordering of the respective tools. We list all paradata and data selection criteria variables in Table 5, all user-entered data variables in Table 6, and finally all result variables in Table 7. Note that a more detailed version of the codebook can be found on ICPSR^27^.

**Table 5:** Codebook for the user datasets - paradata variables and selection criteria variables.

Variable name	Description	Values
**Paradata**	**Automatically collected**	
tool	Identifies the specific VAA	“Region, Newspaper” or “Region, no media outlet”
token	Unique identifier for each user	Alphanumeric string
used_language	Language in which the tool was offered	Language codes (e.g., “en”, “nl”)
date	Date when the user accessed the tool	YYYY-MM-DD
session_start	Precise timestamp when the session began	YYYY-MM-DD HH:MM:SS
before_after_election	Indicates whether the session occurred before or after first election results	“before”/“after”
seconds_spent_answering	Total time (in seconds) spent on the tool	Numeric
seconds_on_statements	Time (in seconds) spent answering issue statements	Numeric
percentage _of_statements_answered	Percentage of issue statements answered	0–100
identical_statement_answers	Number of statements answered with the same response	Numeric
identical_statement_answers_prop	Proportion of statements that were answered in the same way	0–1
device_type	Type of device used	e.g., “desktop”, “mobile”
touch_device	Indicates whether the device was a touch device	TRUE/FALSE
continent	Automatically detected continent	Continent name
country	Automatically detected country	Country name
subdivision	Automatically detected region/state/province	Subnational unit
**Selection criteria**		
crit_identical	TRUE if user answers less than 80% of statements identically	TRUE/FALSE
crit_90pctanswered	TRUE if user answered more than 90% of statements	TRUE/FALSE
crit_lessthan1sec	TRUE if user spent more than 1 second per statement	TRUE/FALSE
crit_genuine (2024 only)	TRUE if user indicated answering genuinely	TRUE/FALSE
crit_all	Combination of the above criteria	TRUE/FALSE

**Table 6:** Codebook for the user datasets - user-supplied data variables.

Variable name	Description	Values
**Background variables**		
bgq_age	Respondent’s age	16–98; 99 or older; Prefer not to say9
bgq_age_cat	Respondent’s age category	16–24; 25–34; 35–44 45–54; 55–64; 65–74; 75–99
bgq_sex	Respondent’s gender	M; F; Other; Prefer not to say
bgq_edu	Respondent’s local education level	Varies by tool; Prefer not to say
bgq_edu_isced	Respondent’s local education level recoded to ISCED levels	0–8
bgq_edu_high	Respondent’s local education level, recoded into higher education or not	0: no higher education; 1: higher education
bgq_geo_country_combi	Respondent’s country, derived from bgq_geo_subdivision if entered, otherwise completed by the automatically collected country variable	
bgq_geo_subdivision	Respondent’s subnational geographic location	Varies by tool; Abroad; Prefer not to say (2024 only)
bgq_vot (2024 only)	Respondent’s vote recall in their last national election	Varies by tool; Prefer not to say
bgq_vot_ches; bgq_vot_cmp; bgq_vot_gps; bgq_vot_vdem (2024 only)	Respondent’s vote recall, recoded to Chapel Hill Expert Survey codes^28^; Comparative Manifesto Project codes^29^; Global Party Survey codes^30^; Varieties of Democracy codes^31^	
**Statements**		
[statement_code] starts with uspr20_ or uspr24_	User’s answer to a specific issue statement, ordered by order in VAA	−2 to 2 (Likert scale); 99 for ‘No Opinion’
**Feeling thermometers**		
fts_harris; fts_trump;fts_biden; fts_dems; fts_reps; fts_usgov; fts_usppl; fts_ru; fts_cn (2024 only)	Feeling thermometer toward Kamala Harris; Donald Trump; Joe Biden; the Democratic Party; the Republican Party; the US government; the American people; Russia; China	0–10; 98: Prefer not to say; 99: No opinion
**Attitudinal items**	**Before result variables in 2024, after result variables in 2020**	
att_lr	Where would you place yourself on a scale from 0 to 10, where 0 is the most economically left-wing and 10 is the most economically right-wing.	0–10; 99: Prefer not to say
att_cp	Where would you place yourself on a scale from 0 to 10, where 0 is the most culturally conservative and 10 is the most culturally liberal.	0–10; 99: Prefer not to say
att_voteintention_usa	If you could vote in the US election, who would you vote for?	2024: Kamala Harris; Donald Trump; Someone else; Would not vote; I prefer not to say
att_dir_usa	Would you say that, overall, things are going in the right or the wrong direction in your own country?	Right direction; Wrong direction
att_dir_own (not in US tool)	Would you say that, overall, things are going in the right or the wrong direction in the US?	Right direction; Wrong direction
att_vot (2020 only)	Respondent’s vote recall in their last national election	Varies by tool
att_vot_ches; att_vot_cmp; att_vot_gps; att_vot_vdem (2020 only)	Respondent’s vote recall, recoded to Chapel Hill Expert Survey codes^28^; Comparative Manifesto Project codes^29^; Global Party Survey codes^30^; Varieties of Democracy codes^31^	
att_voteintention_own (not in US tool, 2020 only)	Respondent’s vote intention if their national election was held today	Varies by country
att_qualification_biden (2020 only)	How qualified do you think Joe Biden is as president for the US?	Very unqualified; Unqualified; Neither qualified nor unqualified; Qualified; Very qualified
att_qualification_trump (2020 only)	How qualified do you think Donald Trump is as president for the US?	Very unqualified; Unqualified; Neither qualified nor unqualified; Qualified; Very qualified
att_goodforusa_trump (2020 only)	On a scale of 0 to 10, how good would you say Donald Trump is for America?	0–10; 99: I don’t know/no opinion
att_amexceptionalism	The US is the greatest country on earth	Strongly disagree; Somewhat disagree; Somewhat agree; Strongly agree
att_govpreference (not in US tool)	Do you think your national government has a preference for who wins the US election? If so, which candidate do you think they prefer?	I think my government prefers Kamala Harris; I think my government prefers Donald Trump; I don’t think my government has a preference; I prefer not to say
att_reliability (only 2024)	While filling out this survey, I was:	Expressing my genuine views; Trying to end up close to Donald Trump; Trying to end up close to Kamala Harris; Just playing around with my answers (e.g. following a pattern or picking randomly); Other

**Table 7:** Codebook for the user datasets - result variables variables.

Results		
pct_biden (2020 only)	Percentage match with Joe Biden	0–100
pct_trump	Percentage match with Donald Trump	0–100
pct_harris (2024 only)	Percentage match with Kamala Harris	0–100
pct_highest	Highest matching candidate	Candidate name or “Equal”
position_x, position_y (2020 only)	User’s calculated position in political landscape	−2 to 2

Note that the 2024 tool for US audiences inadvertently got distributed in the Netherlands, and, therefore, many responses stem from the Netherlands as opposed to from the US. These responses can be recognized by the ‘country’ column.

## Technical Validation

A number of checks can be conducted to validate our data. Starting with the placement data, we investigate the ideological positioning of the candidates. Logically, we would expect both Democratic candidates to be positioned economically left and culturally liberal with respect to Donald Trump. Figure 2 confirms that this is the case. In 2024, we followed a more rigorous coding process that had two coders place both candidates independently. They achieved an intercoder reliability of 0.78 (weighted Cohen’s kappa, ICC, and Krippendorf). Future studies may seek convergent validity e.g. through artificial intelligence coding estimates.

Second, we investigate the consistency of users’ answers. One way we might do that is by looking at the correlation between the tool’s results and users ideological self-placement. In 2020, we can compare with both the users’ percentage agreement scores with the candidates and with the users’ positions in the landscape. The correlation between left-right self-placement is 0.53 (p < 0.001) with ‘position_x’, −0.54 (p < 0.001) with agreement with Biden, and 0.54 (p < 0.001) with agreement with Trump. The correlation between conservative-liberal self-placement is 0.49 (p < 0.001) with ‘position_y’, 0.48 (p < 0.001) with agreement with Biden, and −0.48 (p < 0.001) with agreement with Trump. These patterns are highly similar for all 2020 tools separately as well. In 2024, we can only compare with the agreement scores. The correlation between left-right self-placement is −0.43 (p < 0.001) with agreement with Harris and 0.45 (p < 0.001) with agreement with Trump. The correlation between conservative-liberal self-placement is 0.43 (p < 0.001) with agreement with Harris, and −0.43 (p < 0.001) with agreement with Trump. Similarly, we would expect high correlations between Trump and Harris’ thermometer ratings and the percentage agreement with each. Note that the thermometers were only asked in 2024. The correlation between Trump’s thermometer rating and agreement with Trump is 0.69 (p < 0.001). For Harris, this correlation is 0.67 (p < 0.001). This provides some evidence that the outcome variables of the tools align with users’ ideologies.

Third, to ensure robustness of the results shown to users, we may investigate what proportion of users received results where both candidates are close to each other. Both in 2020 and in 2024, fewer than 1% of respondents received results where agreement with both candidates was equal. Precisely 5% of users in 2020 were shown results where the difference between the candidates was less than 5%. In 2024, 6.4% of users were shown results where the difference between the candidates was less than 5%. This provides evidence that for the overwhelming majority, statement selection did not determine the comparison between the candidates.

Fourth, the number of seconds that users spend answering the tool should never be smaller than the number of seconds they spend on the statements alone. This is true in all tools. On average, users spend 213 seconds on the tool, of which 147 seconds on the issue statements and 66 seconds elsewhere. These durations appear to pass a face validity test.

Lastly, due to the gamified nature of VAAs, we risk that users do not provide genuine answers at higher rates than they might in other surveys. In 2024, we asked users whether they filled out the tool while expressing their genuine views, trying to end up closer to either candidate, or just playing around. Of all respondents who answered this question, 89.5% say they were answering genuinely. Of course, for a user who’s answering randomly, this answer may not be so credible. However, we may investigate the consistency of these users’ answers. We would expect higher internal consistency on both dimensions for the genuine responses compared to the random responses. As expected, genuine answers have a Cronbach’s alpha of 0.44 on the economic dimension and of 0.85 on the cultural dimension, whereas random answers have a Cronbach’s alpha of 0.33 on the economic dimension and of 0.75 on the cultural dimension.

## Usage Notes

To ensure the validity and reliability of the data being used, it is recommended that any records which do not meet any of the first three criteria shown in Table 8 be filtered out.

**Table 8:** Usage notes, selection criteria.

Criterion	Reason	Included in ‘crit_all’
Less than 80% of statement responses are identical across the tool.	Eliminating absent minded answers	Yes
More than 90% of statements answered.	Uniformity and completeness of data	Yes
Time spent on a tool equal to or greater than one second per question.	Thoroughness of responses	Yes
Indication of genuine expression in responding to questions.	Bias mitigation (2024 only)	No

All of these criteria are reflected in their respective variables (see Table 4). The variable ‘crit_all’ combines the top three criteria as recommended, allowing for simple filtering of records. While the genuine response criterion (included in 2024 only) is not independent from the combined criteria (χ²(1) = 82, p < 0.0001), it performs below expectations substantively (Cohen’s Kappa = 0.02). Since the criterion does not feature in the 2020 data, it is not included in the ‘crit_all’ variable, but users may consider using it to filter records, particularly when investigating the 2024 data.

## Data Availability

The data described above is made available on the Inter-university Consortium for Political and Social Research (ICPSR) database [238229]^27^. It includes two datasets comprising the candidates’ placements on 29 statements in 2020 and 62 statements in 2024, as well as two user datasets covering 571,624 user records in 2020 and 152,670 user records in 2024. We also include a codebook for these four datasets. In addition, we include an overview of all statements between both election cycles, and which VAAs these statements were included in.

## Code availability

Replication code and recommended code are available in the ‘code’ folder in the same location as the data files on the ICPSR database^27^.
